# Chronic endometritis and the endometrial microbiota: implications for reproductive success in patients with recurrent implantation failure

**DOI:** 10.1186/s12941-024-00710-6

**Published:** 2024-05-30

**Authors:** Hong Zhang, Heng Zou, Chanyu Zhang, Shen Zhang

**Affiliations:** 1 The Center for Reproductive Medicine, Obstetrics and Gynecology Department, The Second Affiliated Hospital of Chongqing Medical University, Chongqing, 400010 China; 2 Joint International Research Lab for Reproduction and Development of Ministry of Education of China, Chongqing Medical University, Chongqing, 400010 China

**Keywords:** Chronic endometritis, Endometrial microbiota, Recurrent implantation failure, Reproductive outcomes, Microbial diversity

## Abstract

**Background:**

Chronic endometritis (CE) is associated with poor reproductive outcomes, yet the role of endometrial microbiota in patients with recurrent implantation failure (RIF) and CE remains unclear. This study aims to characterize endometrial microbiota in RIF patients with CE and assess its implications for reproductive outcomes.

**Methods:**

In this prospective study, we enrolled RIF patients both with and without CE. Endometrial and cervical samples were collected for 16 S rRNA gene sequencing. Microbiota composition was compared between groups using diversity indices, phylum, and genus-level analysis. Canonical correlation analysis (CCA) and Spearman’s correlation coefficients were used to assess relationships between CE, reproductive outcomes, and microbiota. Predictive functional profiling was performed to evaluate metabolic pathways associated with CE.

**Results:**

Endometrial microbiota in CE patients exhibited greater diversity and evenness compared to non-CE patients. Principal coordinates analysis (PCoA) revealed distinct clustering between CE and non-CE groups. Linear discriminant analysis (LDA) identified *Proteobacteria*, *Aminicenantales*, and *Chloroflexaceae* as characteristic of CE, while *Lactobacillus*, *Acinetobacter*, *Herbaspirillum*, *Ralstonia*, *Shewanela*, and *Micrococcaceae* were associated with non-CE. CCA demonstrated associations between CE, adverse reproductive outcomes, and specific bacterial taxa. Microbial metabolic pathways significantly differed between CE and non-CE groups, with enrichment in pathways related to cofactors, vitamins, secondary metabolites, and the immune system in CE patients.

**Conclusion:**

RIF patients with CE exhibit distinct endometrial microbiota compositions associated with adverse reproductive outcomes. The increased microbial diversity and altered metabolic pathways in CE suggest a potential correlation with reproductive outcomes, although further studies are necessary to elucidate the causal relationship between microbiota alterations and fertility. Modulating the endometrial microbiome may represent a novel therapeutic strategy to improve IVF outcomes in patients with CE.

**Supplementary Information:**

The online version contains supplementary material available at 10.1186/s12941-024-00710-6.

## Background

Recurrent Implantation Failure (RIF) is a distressing condition in reproductive medicine, defined by the inability to achieve clinical pregnancy after the transfer of several good-quality embryos during assisted reproductive technology (ART) procedures. The etiology of RIF is multifaceted, encompassing maternal, paternal, and embryonic factors [1]. These include immunological issues, anatomical abnormalities of the uterus, chromosomal anomalies in embryos, and endometrial receptivity problems. Despite the high quality of embryos transferred, RIF remains a significant obstacle to successful pregnancy. The complexity of RIF underscores the need for a deeper understanding of its underlying mechanisms, particularly in relation to the endometrial environment.

Chronic endometritis (CE) is a disease characterized by persistent low-grade inflammation of the endometrium, primarily attributed to bacterial infection, although hormonal imbalances and autoimmune disorders may also contribute to its development [2, 3]. The prevalence of CE varies widely, ranging from 0.2 to 46%, depending on the patient characteristics and diagnostic methods [3, 4]. Clinical manifestations include pelvic pain, abnormal vaginal bleeding, and vaginitis, although many individuals with CE remain asymptomatic. CE is associated with adverse reproductive outcomes, such as RIF [5, 6]. Antibiotic therapy significantly improves reproductive outcomes in patients with CE [7, 8].

The upper genital tract harbors low-biomass microbiota, and the indigenous endometrial microbiota remains unclear [9–11]. The abundance of *Lactobacillus* in the endometrium is associated with increases in implantation, pregnancy, ongoing pregnancy and live birth [12–16]. CE is significantly correlated with altered endometrial microbiota [17], characterized by higher abundance of certain bacterial taxa and decreased *Lactobacillus*, potentially disrupting microbiota balance and favoring pathogenic bacterial proliferation [18, 19]. This dysbiosis could potentially impact embryo implantation and contribute to poor reproductive outcomes [13]. Moreover, the metabolic pathways of the endometrial microbiota have been found to differ significantly between CE and non-CE patients, indicating potential involvement in CE pathogenesis [19].

Despite these findings, the relationship between the endometrial microbiota, CE, and reproductive outcomes in RIF patients remains unclear. In this study, we aim to address this knowledge gap by characterizing the endometrial microbiota in RIF patients with CE and assessing its implications for reproductive success. We hypothesize that the endometrial microbiota in RIF patients with CE will exhibit distinct characteristics that correlate with adverse reproductive outcomes. Understanding the specific alterations in the microbiota and their functional implications could lead to the development of novel therapeutic interventions aimed at modulating the endometrial microbiome to improve in vitro fertilization (IVF) success rates in individuals with CE.

## Methods

### Study participants

83 asymptomatic women with RIF were recruited in the Center for Reproductive Medicine at the Second Affiliated Hospital of Chongqing Medical University between October 2022 and December 2023 with 6 months of follow-up after the last embryo transfer. RIF was defined as the failure of clinical pregnancy after 4 good quality cleavage stage embryo transfers or 2 good quality blastocyst transfers, with at least three fresh or frozen IVF cycles. Clinical pregnancy was defined as an intrauterine pregnancy up to 12 weeks of gestation. Miscarriage was defined as the loss of a pregnancy before the completion of 12 weeks of gestation. The inclusion and exclusion criteria were modified from a previous study (Supplemental Table S1) [20].

**Fig. 1:**
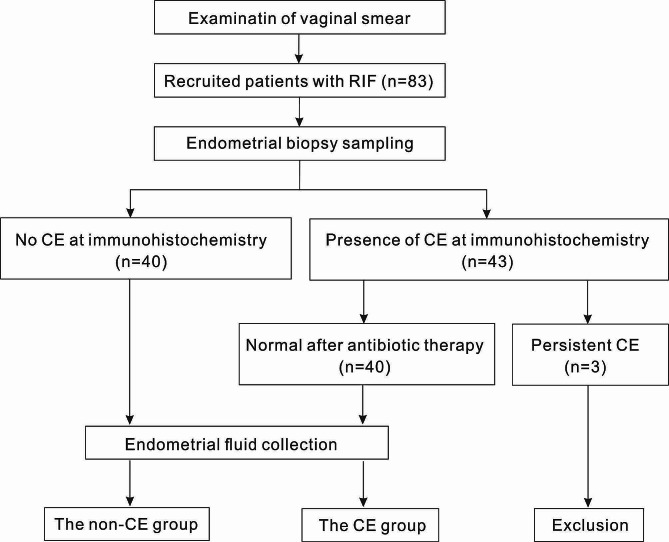
Flow diagram of the study All patients with RIF underwent vaginal smear examination, and those with a dominant *Lactobacillus* flora were selected for inclusion. A total of 83 RIF patients were enrolled. These patients underwent hysteroscopy and endometrial biopsy to confirm the presence of uterine structural normalities and endometritis. No uterine structural abnormalities were detected. Based on the examination results, patients were categorized into endometritis and non-endometritis groups. Endometritis group patients were treated with antibiotics and underwent repeat endometrial biopsy. Three patients with persistent endometritis were excluded. The remaining endometritis and non-endometritis group patients had their endometrial flushing fluid collected for 16 S rRNA sequencing analysis RIF, recurrent implantation failure; CE, chronic endometritis

The clinical characteristics of the study population are detailed in Table 1. The initial cohort of 83 patients with RIF was reduced to 80 for the final analysis, with 40 patients in each of the non-CE and CE groups, as illustrated in Fig. 1. There were no significant differences between the groups in terms of age, BMI, duration of infertility, history of previous conception, AMH level, basal FSH level, basal LH level, basal E2 level, basal P level, and basal PRL (*P* > 0.05).

**Table 1:** Clinical characteristics of the study population

Variables	Non-CE group (*n* = 40)	CE group(*n* = 40)	*P* value
Age (years)	32.88 ± 2.03	30.57 ± 2.64	0.08^a^
BMI (kg/m^2^)	21.11 ± 1.75	19.96 ± 1.61	0.19^a^
Duration of infertility (years)	2 (1, 2)	2 (1, 2)	0.95^b^
Previous conception	0 (0, 1)	0 (0, 1)	0.90^b^
AMH (mIU/ml)	3.01 (2.08, 6.83)	3.99 (2.10, 6.03)	1.00^b^
Basal FSH (mIU/ml)	6.36 (4.36, 7.11)	6.50 (5.83, 7.12)	0.76^b^
Basal LH (mIU/ml)	5.01 (1.41, 0.64)	5.43 (1.96, 5.00)	0.64^b^
Basal E2 (pg/ml)	38.62 (35.00, 49.99)	35.72 (27.37, 43.28)	0.47^b^
Basal P (ng/ml)	0.34 ± 0.23	0.24 ± 0.15	0.33^a^
Basal PRL (ug/L)	20.58 ± 7.74	18.30 ± 4.74	0.16^a^
CD 138^+^ (/HPF^+^)	0 (0, 0)	10 (5, 15)	0.00 ^b^
Histological findings	Normal (40/40)	Plasma cell infiltration (40/40)	0.00 ^c^
Previous antibiotics treatment	0 (0, 0)	0 (0, 1)	0.00 ^b^

Abbreviations: FSH (follicle-stimulating hormone); LH (luteinizing hormone); E2 (estrogen); PRL (prolactin); AMH (anti -mullerian hormone)

^a^Student t-test. The data were presented as the mean ± SD

^b^Mann-Whitney U-test. The data were presented as the median (25th percentile, 75th percentile)

^c^Chi-square test

### Examination of vaginal smear

During speculum examination, vaginal swabs were collected and smeared on glass-slides. Then, the slides were stained with Gram’s method and observed by oil lens (1000×). The Nugent scoring was performed according to a standard protocol [21]. Large gram-positive rods were considered as *Lactobacillus*.

### Diagnosis and management of CE

At present, there is no universally accepted consensus on the diagnosis of CE. The diagnosis of CE in this study was based on a combination of hysteroscopy and immunohistochemistry, as previously described [22, 23]. For the clinical management of CE, particularly in the context of patients with RIF, it is essential to identify the underlying causes of implantation failure. This was achieved through hysteroscopy, which allowed for the examination of abnormal uterine shape or structure and the detection of chronic endometritis. Patients without structural abnormalities were included in the study. Following this initial assessment, endometrial biopsy was conducted to obtain a tissue sample for histopathological examination. This examination was then performed to confirm the presence of CE.

### Endometrial biopsy sampling and immunohistochemistry for CD138

Endometrial biopsy was performed in the follicular phase as described previously [24]. Endometrial samples were fixed in neutral formalin and embedded in paraffin for immunohistochemistry. All biopsy blocks were serially sectioned at a thickness of 6 μm and incubated with rabbit anti-human monoclonal CD138 antibody (10593-1-AP, Proteintech, No.666, Gaoxin Avenue D3-3, Wuhan, China) and the secondary antibody used was a horseradish peroxidas-conjugated affinipure goat anti-rabbit IgG (PR30011, Proteintech) following the protocol. Immunorecative signals were visualized and photographed with a Nikon DS-Fi3 camera on a Nikon E200 microscope (Nikon, Shinagawa Intercity Tower C, 2-15-3, Konan, Minato-ku, Tokyo, Japan). The number of CD138^+^ cells more than 4/HPF^+^ was diagnosed as CE [23].

### Treatment of CE

In case CE diagnosis, Doxycycline (Jiangsu Lianhua Pharmaceutical Co., LTD, No.21, Wenfeng Road, Yangzhou, Jiangsu, China) 100 mg twice a day for 14 days was employed. In the follicular phase of the cycle following the therapy, endometrial biopsy and were immunohistochemistry for CD138 repeated. Three patients were excluded for persistent CE in the following analysis.

### Cervical mucus and endometrial fluid Collection

Cervical mucus and endometrial fluid were collected from the same patient seven days after the luteinizing hormone surge in natural cycles. The perineum was cleaned by cotton swabs soaked in iodophor solution (Shandong Lilkang Medical Technology Co. Ltd, No.1, Lierkang Road, Dezhou, Shandong, China) with the patient in classic lithotomy position. After inserting a vaginal speculum, the vaginal secretions were removed by cotton swabs soaked in saline solution. Cervical samples were obtained using nylon flocked swabs. After removal of cervical mucus, endometrial fluid were collected with a double-lumen embryo transfer catheter (T-1,731,511, Pacific Contrast Scientific Instruments Co. Ltd, No.1777,Dazheng road, Jinan, Shandong, China) as previously described [17]. Briefly, the outer sheath of the catheter was inserted into the endocervix avoiding contact with the vaginal wall. Subsequently, the inner catheter was inserted into the sheath and advanced into the uterine cavity. 1.0 mL of saline solution was injected and withdrawn to collect the endometrial flushing fluid. After the inner catheter was re-sheathed, both the sheath and catheter were withdrawn from the uterine cavity.

### DNA extraction

DNA isolation was performed as described previously [25]. Endometrial fluid samples were pre-treated with lysozyme (9001-63-2, Sigma-Aldrich, PO Box 14,508, St. Louis, MO, USA), lysostaphin (9011-93-2, Sigma-Aldrich), and mutanolysin (55466-22-3, Sigma-Aldrich). Total DNA was further extracted using a QIAamp DNA Blood Mini kit (Qiagen, Strasse 1, Hilden, Germany) according to the manufacturer’s instructions. The genomic DNA was quantified by NanoDrop 2000 (Thermo Scientific, 168 3rd Ave, Waltham, MA, USA) and its integrity was assessed by agarose gel electrophoresis. Additionally, no-template controls (NTCs) were included in the DNA extraction process to confirm the absence of contaminants from the extraction kit.

### Polymerase chain reaction and 16 S ribosomal RNA gene sequencing

The V3-V4 region of the 16S rRNA gene was amplified by PCR with the barcode-index primers 338F (5’-ACTCCTACGGGAGGCAGCAG-3’) and 806R (5’-GGACTACHVGGGTWTCTAAT-3’) using a TransStart FastPfu DNA polymerase (TransGen Biotech, No.1 Yongtaizhuang North Rd, Beijing, China) on a GeneAmp 9700 thermocycler (Applied Biosystems, 42 North Rd, Wakefield, RI, USA). PCR reactions were performed as following program: 3 min of denaturation at 95℃; 25 cycles of denaturation at 95℃ for 30 s, annealing at 55℃ for 30 s, and elongation at 72℃ for 45 s; and a single extension at 72℃ for 10 min. To confirm the absence of contaminants, we included NTCs in the PCR amplification. The absence of amplification in the NTCs indicated that the PCR reagents were free from contaminants. The PCR products were recovered from a 2% agarose gel by AxyPrep DNA Gel Extraction Kit (Axygen Biosciences, 33,170 Central Ave, Union City, CA, USA). The quality and concentration of the purified amplicons were assessed by a Quantus™ Fluorometer (Promega, 2800 Woods Hollow Rd, Madison, WI, USA). Purified amplicons were pooled in equimolar amounts. Then, the library was constructed using NEXTFLEX Rapid DNA-Seq Kit (PerkinElmer, 940 Winter St, Waltham, MA, USA) following the manufacturer’s instruction. Paired-end sequencing was performed using a NovaSeq system (Illumina, 5200 Illumina Way, San Diego, CA) with PE250 platform.

### Sequencing data analysis

Sequencing reads were treated for quality control and length-filter using fastp (0.19.6) [26]. Taxonomic assignment was performed following QIME2 pipeline [27]. Paired-end reads were merged by Fast Length Adjustment of Short reads (FLASh) tool and de-noised using DADA2 [28, 29]. The amplicon sequence variants (ASVs) generated by DADA2 were used for taxonomic assignment by naive Bayes classifier based on aligning the representative sequences to the SILVA rRNA database (SSU 138 release) [30].

Linear discriminant analysis effect size was used with the parameters (α = 0.05 and LDA score 3.0) to identify any genera that differential in relative abundance between the two groups [31].

## Results

### Differences between endometrial and cervical microbiota

**Fig. 2:**
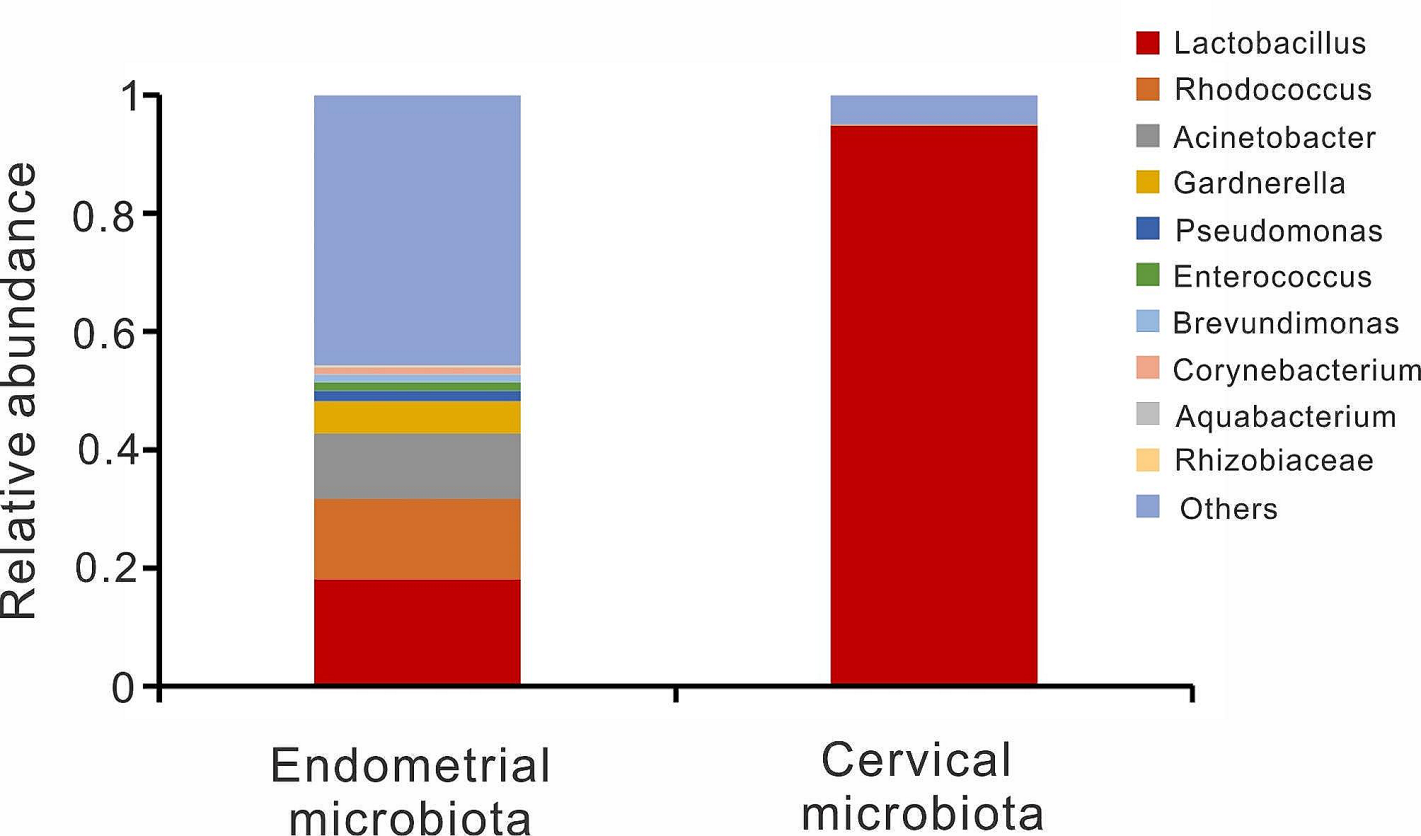
Bar charts showing mean values of 10 most abundant genera in endometrium and cervix of 20 paired samples from 10 enrolled patients. Analysis and plotting were performed using R software (version 3.3.1)

The human vaginal microbiota plays a critical role in preventing several urogenital diseases [32–34]. To minimize the potential influence of vaginal microbiota on reproductive outcomes, we included patients with *Lactobacillus*-dominated vaginal microbiota. To investigate the presence of different microbiota between endometrial and cervical microbiota, paired samples of endometrial fluid and cervical mucus were collected for 16 S rRNA gene sequencing. Distinct microbiota profiles were identified between endometrial and cervical samples (Fig. 2). Cervical microbtiota was dominated by *Lactobacillus*, while endometrial microbiota was non-*Lactobacillus*-dominated.

### Comparison of endometrial microbiota composition in the Non-CE and CE groups

**Fig. 3:**
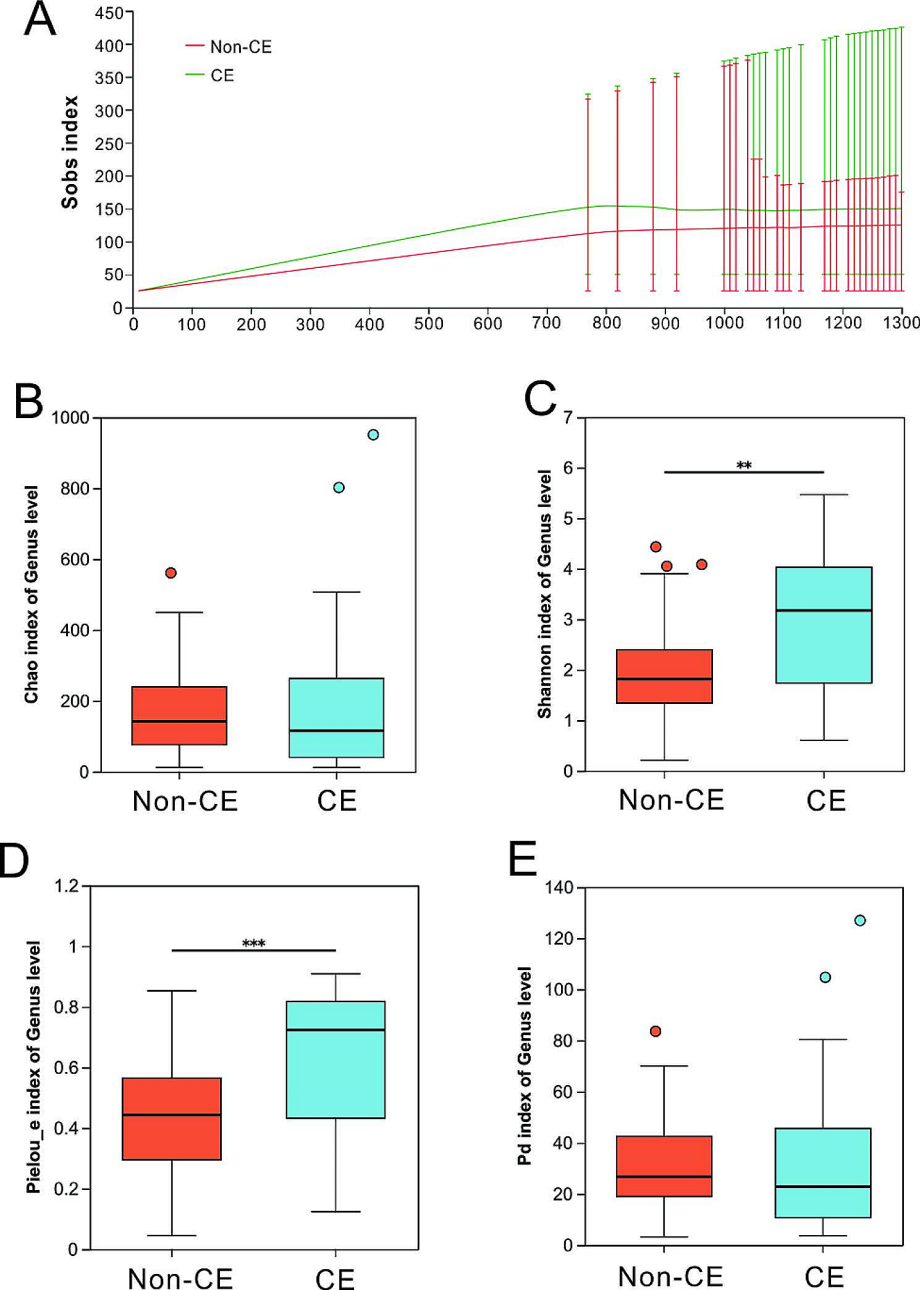
Microbial diversity in the non-CE and CE groups (**A**) Rarefaction curve for observed species (Sob). Comparison of Chao index (**B**), Shannon index (**C**), Pieou’s evenness index (**D**) and Phylogenetic diversity (Pd) index (**E**) between the non-CE and CE groups. Statistical analysis was performed using mothur software (version v.1.30.2), and plotting was performed using R software (version 3.3.1). Data were represented as mean ± SD, *n* = 40. *P < 0.05, **P < 0.01, ***P < 0.001 by two-tailed Wilcoxon rank-sum tests followed by FDR corrections

To avoid contact with the vaginal and cervical wall, a double-lumen embryo transfer catheter with an outer sheath was used to collect endometrial microbiota. Rarefaction curve for observed species (Sobs) was used to assess the saturation of the sample size (Fig. 3A). This analysis confirmed that the 16 S rRNA gene sequencing in our study achieved adequate sequencing depth. Chao richness (*P* = 0.4443) and phylogenetic diversity (Pd, *P* = 0.3963) did not significantly differ between non-CE and CE patients (3B and 3E). However, Shannon diversity (*P* = 0.0026) and Pielou’s evenness (*P* = 0.0003) were significantly greater in samples from CE patients versus non-CE patients (Fig. 3C-D). These results indicate an increase in endometrial microbial diversity and evenness in CE patients.

**Fig. 4:**
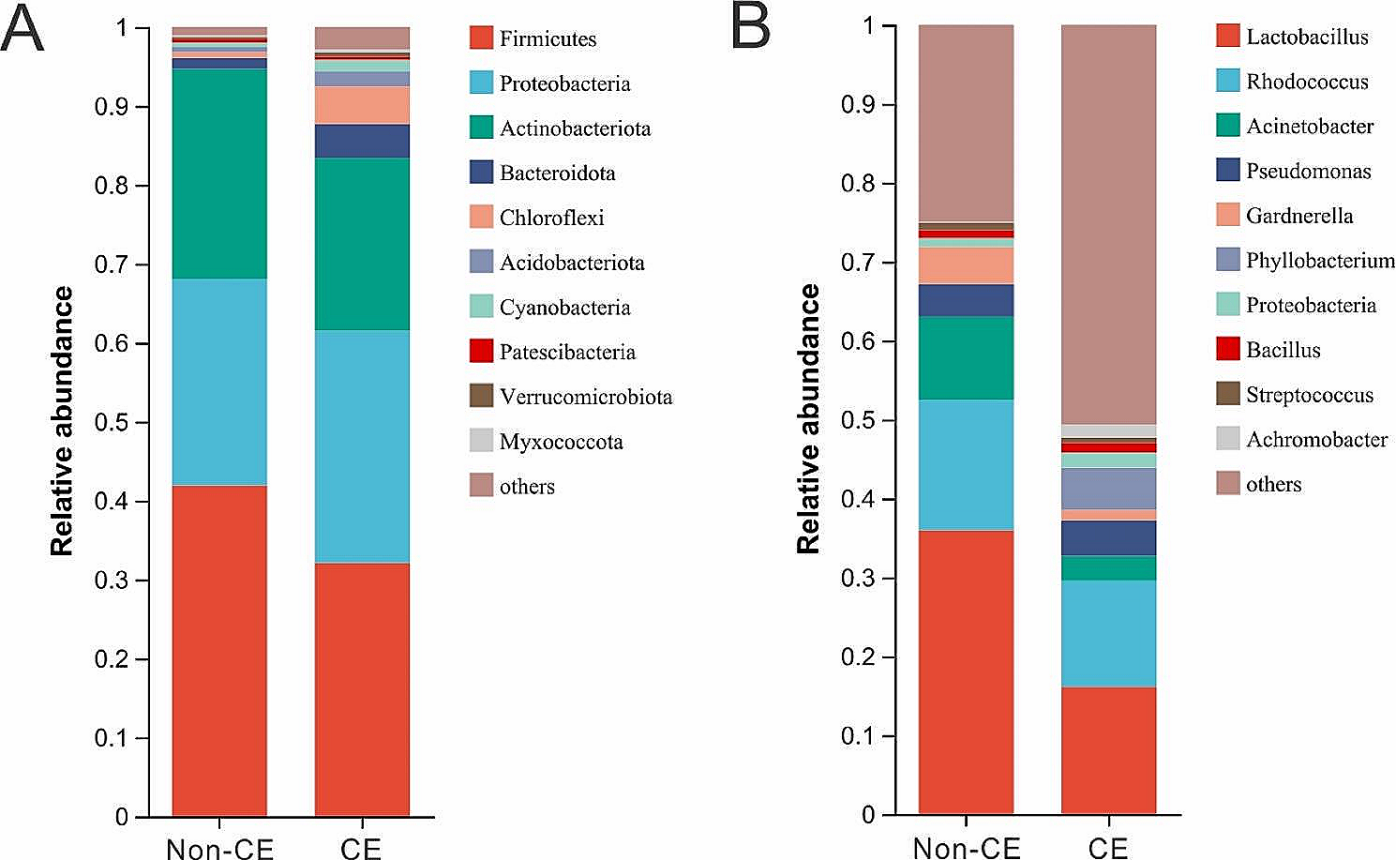
Bacterial communities in endometrial microbiota of the non-CE and CE groups Bar charts showing showing mean values of 10 most abundant phyla (**A**) and genera (**B**) in the non-CE and CE groups. Analysis and plotting were performed using R software (version 3.3.1)

**Fig. 5:**
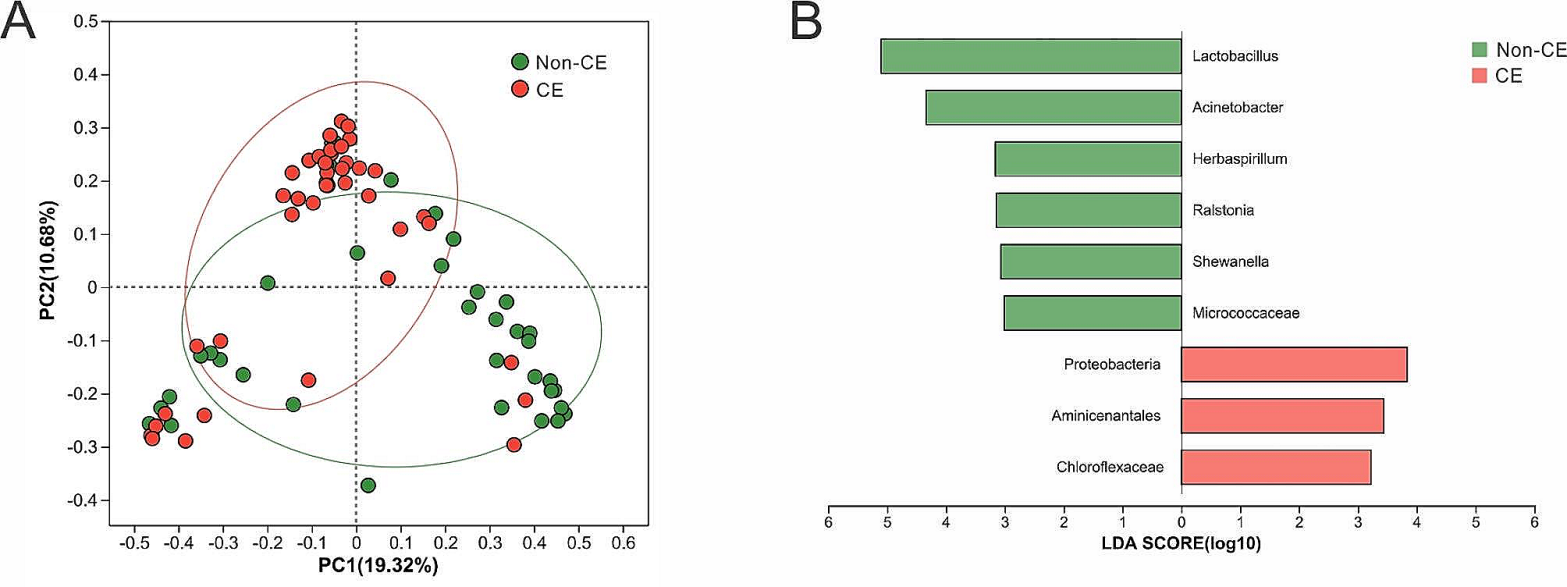
Identification of the discriminatory genera (**A**) PCoA plots of uterine samples from the non-CE and CE patients. R software (version 3.3.1) was used for PCoA analysis and visualization (**B**) Linear discriminant analysis (LDA) effect size analysis of the differentially abundant genera, which indicated their contribution to group differentiation. The green bars indicate that the genera were more abundant in the non-CE group, while the red bar indicates that the genus was more abundant in the CE group. LEfSe analysis was employed to confirm the significance of the LDA-selected genera, ensuring that only those with the strongest contribution to group separation were identified

At the phylum level, *Firmicutes*, *Actinobacteriota* and *Proteobacteria* collectively accounted for over 80% of the microbiota (Fig. 4A). No dominant phylum was identified. At genus level, the top microbiota was *Lactobacillus*, *Rhodococcus*, *Acinetobacter* and *Pseudomonas* (Fig. 4B). Principal coordinates analysis (PCoA) based on Bray-Curtis distances at genus-level revealed a statistical separation between the non-CE and CE groups (*P* = 0.0010, Adonis test) (Fig. 5A). Linear discriminant analysis (LDA) effect size was applied to identify statistically significant genera associated with each group. *Proteobacteria* (*P* = 0.0315), *Aminicenantales* (*P* = 0.0467) and *Chloroflexaceae* (*P* = 0.0235) were associated with CE, while *Lactobacillus* (*P* = 0.0007), *Acinetobacter* (*P* = 0.0051), *Herbaspirillum* (*P* = 0.0081), *Ralstonia* (*P* = 0.0106), *Shewanela* (*P* = 0.0258) and *Micrococcaceae* (*P* = 0.0247) were characteristic of the non-CE group (Fig. 5B). Our results revealed significant differences between the endometrial bacterial communities of the non-CE and CE groups.

### Endometrial microbiota composition and reproductive outcomes

**Fig. 6:**
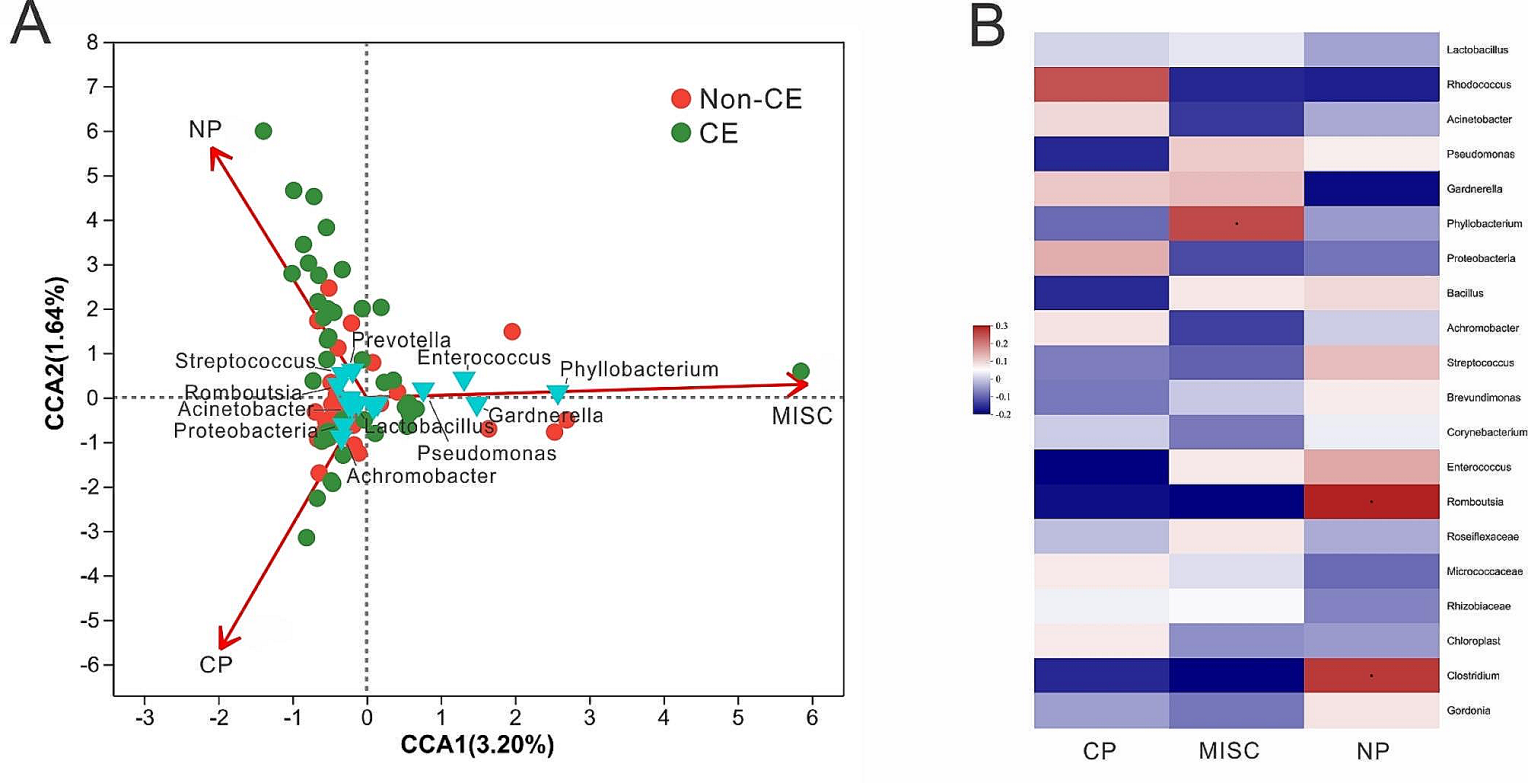
Relationships between endometrial microbiota and reproductive outcomes (**A**) Canonical correspondence analysis (CCA) diagram showing the associations between CE, endometrial microbiota and reproductive outcomes. Vegan package (version 2.4.3) in R software (version 3.3.1) was used for the CCA analysis and visualization. Statistical significance was determined by *P* values: clinical pregnancy (CP, *P* = 0.01), miscarriage (MISC, *P* = 0.01), and non-pregnancy (NP, *P* = 0.01) (**B**) Spearman correlation heatmap analysis between endometrial microbiota and reproductive outcomes. Red represents a positive correlation, and blue represents a negative correlation. The heatmap was generated using the pheatmap (version 1.0.8) package in R software (version 3.3.1)

Following antibiotic treatment for CE, reproductive outcomes were compared between the non-CE and CE groups. The clinical pregnancy rate was significantly higher in the non-CE group, whereas the miscarriage rate showed no difference between the two groups (Table 2). Canonical correlation analysis (CCA) showed that CE was related to adverse reproductive outcomes and endometrial microbiota (Fig. 6A). Specifically, *Phyllobacterium*, *Gardnerella*, *Enterococus* and *Pseudomonas* were correlated with miscarriage. *Achromobacter*, *Proteobacteria*, *Lactobacillus* and *Acinetobacter* were associated with clinical pregnancy, while *Prevotella*, *Streptococcus* and *Romboutsia* were correlated with non-pregnancy. Spearman’s correlation coefficients also revealed that *Phyllobacterium* (*P* = 0.0429) were characteristic of miscarriage, while *Romboutsia* (*P* = 0.0167) and *Clostridium* (*P* = 0.0297) were associated with non-pregnancy (Fig. 6B).

**Table 2:** Comparison of reproductive outcomes between the non-CE and CE groups

Reproductive outcomes	Non-CE group (*n* = 40)	CE group (*n* = 40)	*P* value	Odds ratio	95% Confidence Interval
Clinical pregnancy rate	62.5% (25/40)	37.5% (15/40)	0.04	2.78	1.12; 6.87
Miscarriage rate	13.8% (4/29)	21.1% (4/19)	0.79	0.60	0.13; 2.76

### Microbial metabolic pathways significantly associated with CE

**Fig. 7:**
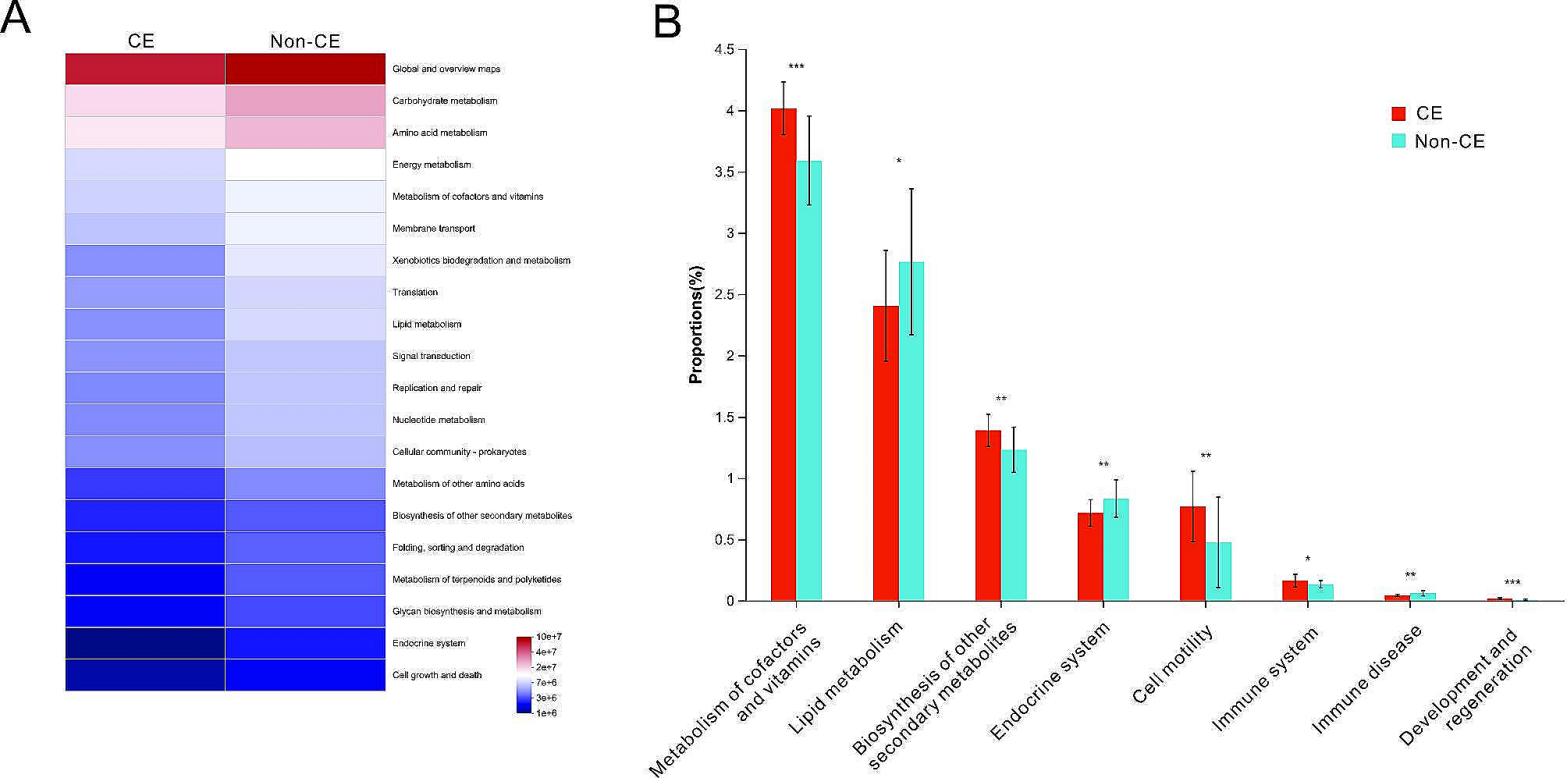
The functional properties predicted by PICRUSt2 (**B**) (A) Heatmap showing the predicted microbial metabolism pathways of the endometrial microbiota of the CE and non-CE groups. Relative proportions of the predicted functions with significant difference between the two groups. *P < 0.05, **P < 0.01, ***P < 0.001 by two-tailed Wilcoxon rank-sum tests followed by FDR corrections The functional analysis was performed using PICRUSt2 software

The functional properties of genera detected in 16 S rRNA gene analysis were predicted with PICRUSt2. The metabolic pathways were found to be notably different between the two groups (Fig. 7A), indicating that the distinction of microbiota caused the difference in the function. Statistically, the pathways including metabolism of cofactors and vitamins (*P* = 0.0003), biosynthesis of other secondary metabolites (*P* = 0.0032) and immune system (*P* = 0.0496) were significantly enriched in the CE group, compared with the non-CE group (Fig. 7B). While lipid metabolism (*P* = 0.0459) and endocrine system (*P* = 0.0064) were significantly decreased in the CE group (Fig. 7B).

## Discussion

This study aimed to investigate the relationship between the endometrial microbiota and reproductive outcomes, particularly in the context of CE and RIF. To our knowledge, it is the first study to elucidate the distinct composition of endometrial microbiota in RIF patients with CE, highlighting its potential correlation with reproductive outcomes, although further studies are necessary to elucidate the causal relationship between microbiota alterations and fertility. Utilizing 16 S rRNA gene sequencing, we compared the microbiota in endometrial fluid of RIF patients with and without CE, revealing significant compositional differences. Notably, we observed a significant increase in microbial diversity and evenness in the endometrial microbiota of CE patients, along with significant changes in microbial metabolic pathways associated with CE.

By collecting the endometrial microbiota using a double-lumen embryo transfer catheter, we minimized the possibility of sample contamination, ensuring the accuracy of our results. Consistent with the previous reports, the cervical microbiota was dominated by *Firmicutes*/*Lactobacillus*, while the proportion of *Actinobacteriota*, *Proteobacteria* and *Bacteroidota* were substantially increased in the endometrium [9, 18]. Our findings support the notion that the endometrial microbiota is not predominantly composed of *Lactobacillus* [9, 11, 35, 36].

Our findings align with those of Bednarska-Czerwińska et al., which demonstrated the dynamics of microbiome changes in the endometrium and uterine cervix during embryo implantation [37]. The predominance of *Lactobacillus* and the negative impact of *Escherichia coli* and *Gardnerella vaginalis* on fertility outcomes in their study reinforce the importance of a balanced endometrial microbiota for successful implantation. Additionally, another previous study identified both physiological and pathological microflora in the endometrium and cervix of women undergoing IVF, underscoring the potential influence of these microorganisms on reproductive success [20].

This study sheds light on the intricate relationship between CE, endometrial microbiota composition, and reproductive outcomes in RIF patients. The observed increase in endometrial microbiota diversity and altered taxonomic composition in CE patients aligns with previous studies [18, 38]. We identified specific bacterial taxa, such as *Phyllobacterium*, *Gardnerella*, *Enterococcus*, and *Pseudomonas*, that were correlated with miscarriage, while *Prevotella*, *Streptococcus*, and *Romboutsia* were associated with non-pregnancy. Notably, these taxa have been previously reported to be more abundant in the CE microbiota [17, 19, 39]. Particularly, *Enterococus* and *Streptococcus*, known infectious agents, may contribute to CE [40].

The association between CE and adverse reproductive outcomes, coupled with the identification of specific bacterial taxa characteristic of CE, underscores the potential clinical significance of endometrial microbiota in RIF patients. These findings suggest that dysbiosis in the endometrial microbiome may contribute to impaired reproductive success in CE patients, possibly through mechanisms involving inflammation, immune dysregulation, and altered metabolic pathways [18, 19].

The microbiota may play a role in the morphological changes of mucosal cells, which has implications for decidualization [41]. Commensal microbiota have been shown to provide protection against pathogenic species, thereby contributing to uterine health and the development of a receptive endometrium [13]. In addition to the bacterial taxa identified in our study, the work by Chen et al. supports the hypothesis that CE endometrial microbiota may regulate immune cells by interfering with carbohydrate and fat metabolism [19]. The study also suggests that CE endometrial microbiota might regulate the Th17 response and the ratio of Th1 to Th17 through lipopolysaccharide (LPS), indicating a potential mechanism for immune modulation by the microbiota. The enrichment of certain metabolic pathways in CE patients, such as those related to cofactors, vitamins, and the immune system, warrants further investigation to elucidate their role in CE pathogenesis and reproductive outcomes. Our study and the findings from Chen et al. collectively suggest that the endometrial microbiota plays a critical role in the complex interplay between CE, the immune system, and reproductive success.

Furthermore, while 16 S rRNA gene sequencing provides valuable taxonomic information, it may not capture the full spectrum of microbial diversity or accurately reflect functional capacity. This is particularly important in the context of low-biomass microbial communities, such as those found in the endometrium. Future studies using shotgun metagenomics, metatranscriptomics, or metabolomics could provide deeper insights into the functional potential and metabolic pathways of the endometrial microbiota, which may offer additional understanding of its role in reproductive outcomes.

While this study provides valuable insights into the relationship between endometrial microbiota, CE, and reproductive outcomes in patients with RIF, several limitations should be acknowledged. Firstly, the sample size, although adequate for the present analysis, may limit the generalizability of the findings to broader populations. Larger, multicenter studies are warranted to validate the observed associations across diverse patient cohorts. Additionally, the study design, being observational, inherently poses the risk of confounding factors influencing the results. While efforts were made to control for potential confounders, such as age, BMI, and duration of infertility, residual confounding cannot be entirely ruled out. Furthermore, the methodology employed in microbiota analysis, while robust, has inherent limitations. The use of 16 S rRNA gene sequencing provides valuable taxonomic information; however, it may not capture the full spectrum of microbial diversity or accurately reflect functional capacity. Moreover, while efforts were made to minimize contamination during sample collection and processing, the potential for environmental contamination cannot be entirely eliminated.

## Conclusions

The findings of this study highlight the significant impact of CE on the composition and diversity of endometrial microbiota in patients with RIF. The increased microbial diversity and altered taxonomic composition observed in CE patients suggest a potential role in influencing reproductive outcomes. Specifically, the association between CE and adverse reproductive outcomes, coupled with the identification of specific bacterial taxa characteristic of CE, underscores the importance of considering endometrial microbiota in the management of RIF patients. Modulating the endometrial microbiome may offer a novel therapeutic approach to improve IVF success rates in this population. However, while the differences in microbiota diversity and composition between CE and non-CE patients are clear, the functional implications of these differences are not yet fully understood and warrant further investigation. Further research is warranted to elucidate the underlying mechanisms and to explore targeted interventions aimed at restoring microbial balance and improving reproductive outcomes in RIF patients with CE.

### Electronic supplementary material

Below is the link to the electronic supplementary material.

Supplementary Material 1

## Data Availability

The datasets used during the current study are available from the corresponding author on reasonable request.

## Abbreviations

ART: Assisted reproductive technology
ASV: Amplicon sequence variants
CCA: Canonical correlation analysis
CE: Chronic endometritis
FLASh: Fast Length Adjustment of Short reads
IVF: In vitro fertilization
LDA: Linear discriminant analysis
Pd: Phylogenetic diversity
PCoA: Principal coordinates analysis
RIF: Recurrent implantation failure
Sobs: Observed species
