# Supplementary material for: Chronic endometritis and the endometrial microbiota: implications for reproductive success in patients with recurrent implantation failure
Source: Ann Clin Microbiol Antimicrob. 2024 May 30;23:49. doi: 10.1186/s12941-024-00710-6 (PMC11140900; doi:10.1186/s12941-024-00710-6)
Supplement: Supplementary file 1 — Supplementary Material 1 [file 12941_2024_710_MOESM1_ESM.docx]

**Supplemental Table S1. Inclusion and exclusion criteria for the study**

| Inclusion Criteria | Exclusion Criteria |
| --- | --- |
| Infertility as defined by World Health Organization | American Fertility Score III/IV and pre-treatment with a gonadotrophin-releasing hormone analog |
| Written informed consent | No written consent |
| Age 25-35 | Age <25 or >35 |
| Qualifcation and performance of the IVF procedure | Disqualification and exclusion from the IVF procedure in accordance with the criteria and qualifications |
| Asymptomatic women diagnosed with RIF | No diagnosed with RIF |
| Body mass index (BMI) between 18.5 and 25 kg/m² | BMI <18.5 or >25 |
| Nonsmokers | Smokers |
| Presence of a normal uterus confirmed by gynecological examination and ultrasound | Presence of an abnormal uterus |
| Vaginal microbiota dominated by *Lactobacillus*, as determined by microbiological assessment | Vaginal microbiota not dominated by *Lactobacillus* |
| No vaginal infections | Vaginal infections |
| No use of antibiotics in one month before the study enrollment | Use of antibiotics within the month preceding the study enrollment |
| No systemic disease | History or presence of systemic diseases |
| No endometriosis | Endometriosis |
| Normal serum prolactin levels | Abnormal serum prolactin levels |
| No history of long-term medication use | History of long-term medication use |
| No presence or history of neoplastic | Presence or history of neoplastic |
| No mental or emotional disorders | Mental or emotional disorders |
| Mongolian race | Race other than Mongolian |
| No use of hormonal contraceptives in 3 months before the study enrollment | Use of hormonal contraceptives in 3 months before the study enrollment |
